# Low-Cyclic Reversed Loading Tests on Full-Scale Precast Concrete Composite Wall Connected by Tooth Groove and Grouted Sleeve

**DOI:** 10.3390/ma17020476

**Published:** 2024-01-19

**Authors:** Xiaoyong Luo, Qi Chen, Chao Deng, Wangcheng Luo, Yang He

**Affiliations:** 1 College of Civil Engineering, Central South University, Changsha 410075, China; csu-luoxy@csu.edu.cn (X.L.); dcxtuseu@163.com (C.D.); wangchluo@163.com (W.L.); tmgc1he@163.com (Y.H.); 2 Engineering Technology Research Center for Prefabricated Construction Industrialization of Hunan Province, Changsha 410075, China

**Keywords:** precast concrete composite wall, low-cyclic reversed loading test, seismic performance, tooth groove and grouted sleeve connection, XPS insulation layer

## Abstract

In this paper, a novel precast concrete composite wall connected by tooth groove and grouted sleeve was introduced, which is produced in factories by means of structure-insulation integrated prefabrication, and the prefabrication and assembly process were presented minutely. To verify the feasibility and reliability of this novel tooth groove and grouted sleeve connection method and explore the joint connection performance and the seismic performance of the precast concrete composite wall connected by tooth groove and grouted sleeve, low-cyclic reversed loading tests with an axial compressive ratio of 0.1 were performed on two full-scale precast concrete composite walls. Moreover, the failure mode, hysteretic curve, skeleton curve, stiffness degradation, displacement ductility, energy dissipation capacity, and reinforcement strain were comprehensively discussed. The research results showed that under the vertical axial load and low-cyclic reversed load, the distributed reinforcements in the wall panel only played a structural role, while the connecting reinforcements at horizontal joints can always effectively transfer stress without bond failure, and the tooth groove and grouted sleeve connection performance was reliable. In addition, the hysteretic curves of the precast concrete composite wall connected by tooth groove and grouted sleeve were full, showing good ductile deformation capacity and energy dissipation capacity. In general, the precast concrete composite wall connected by tooth groove and grouted sleeve not only possessed favorable seismic performance but also showed obvious advantages such as green energy saving, high assembly rate, and less on-site wet operation, which can be applied to practical engineering under reasonable design.

## 1. Introduction

With the accelerated development of the construction industrialization process, the application and promotion of assembled concrete structures has become increasingly widespread [1,2,3,4], and the promulgation and implementation of various standards have also provided technical support for its promotion in terms of theory, design, construction, and acceptance. Among them, the assembled concrete composite structure system is a new system of prefabricated building structures with low energy dissipation, ecological environmental protection, energy saving, and heat preservation [5,6,7,8,9]. Driven by the concept of carbon peaking and carbon neutrality goals, the proposal and application of assembled concrete composite structure systems further highlight the advantages of green, energy-saving and low-carbon industrialized building [10,11,12]. In recent years, innovative precast concrete composite structures such as precast concrete sandwich structures [13,14,15], steel-concrete composite structural walls with internal bracings [16], ceramsite concrete hollow block infilling wall structures [17], polystyrene plate sandwich concrete infilling structures [17,18,19,20], and precast concrete wall with X-shaped steel plate bracings [21] have flourished. The above precast concrete composite structures are all assembled by precast concrete components through horizontal joints and vertical joints [22,23,24,25,26,27]. A large number of studies have shown that the horizontal joint connection technology of precast concrete walls is the key to improving the behavior of assembled concrete composite structures [13,28].

Previously, a large number of novel connection technologies for precast concrete walls have been developed and investigated [29,30,31,32,33,34]. Semelawy et al. [35] introduced an innovative precast wall connected by the precast panels and threaded steel anchor bolts, and experimental research was carried out on it. The results demonstrated that the innovative precast wall had good deformation performance, but the steel anchor bolts at the joint showed yield failure. Sun et al. [36] performed quasi-static loading tests on the new-type precast wall connected by bolts and steel frames to discuss the failure mode, displacement ductility, and hysteretic behavior. And through the test, it was found that the new-type joint connection was reliable due to the high strength of the connectors, and the specimens using this connection method had excellent lateral resistance and superior energy dissipation performance. Fu et al. [37] explored the seismic performance of a new prefabricated shear wall connected by steel plate and bolts and found that the connecting steel plate and the bolt did not yield during the test, and the new connection method was reliable. In addition, the failure mode and energy dissipation capacity of this new prefabricated shear wall were basically the same as the ordinary cast-in-place shear wall. Shen et al. [38] found that this novel precast wall with steel shear key connection showed satisfactory bearing capacity, stiffness, and deformation capacity, and its energy dissipation capacity was slightly better than that of ordinary shear walls. Fan et al. [39] investigated the seismic behaviors of the hybrid braced precast wall with replaceable U-shaped flexural plates and found that the proposed precast wall showed satisfactory energy dissipation, excellent bearing capacity, and low damage abilities. The above new precast walls were all connected by bolts, steel plates, or steel connectors, it can be found that the bolts or steel plates of this connection method can effectively transfer stress at the joints, and the connection was reasonable. Furthermore, the prefabricated walls connected by bolts, steel plates, or steel connectors had similar seismic behavior to ordinary prefabricated walls, and their seismic performance was excellent. However, this connection method had problems such as bolt slip, easy leakage at the joint, complex configuration, and high accuracy construction requirements, which make it difficult to maximize the advantages of assembled prefabricated concrete structures.

Additionally, some scholars have also proposed and studied other novel joint connection technologies. Yuan et al. [40] designed seven new prefabricated shear walls with mortar connection, and concluded that due to the discontinuity of the vertical reinforcements, the mortar layer of the specimens was destroyed under low-cyclic reversed load, accompanied by local buckling of some reinforcements. On the whole, the bearing capacity of the new prefabricated wall has decreased. Jia et al. [41] and Wu et al. [42] investigated the seismic performance of the precast shear wall with a grouting sleeve connection, and it was found that the bond behavior between the connecting steel bars and the grouting materials was degraded after yielding. But overall, this new precast shear wall with grouting sleeve had sufficient displacement ductility and seismic performance. Liu et al. [43] explored the effect of the axial compression ratio on the performance of novel mortise-tenon joints for prefabricated shear walls. Although the mortise-tenon joint was damaged, the deformation capacity of the joint was strong and the joint configuration was reasonable. Furthermore, the specimens exhibited good bearing capacity and deformability under an axial compressive ratio. Feng et al. [44] put forward a novel joint connection of the bundled vertical steel bars in the preformed holes with high-performance grouting material and conducted an experimental study on eight full-scale specimens. It can be concluded that the crack development modes of the novel precast shear wall with bundled vertical steel bars connection was mainly the horizontal crack penetration at the interface between the wall panel and the basement, and most of the cracks were concentrated near the preformed holes. In general, the bundled vertical steel bars connection was an effective connection method. Zhang et al. [45] put forward a new type of precast hollow shear wall and studied the reliability of the connection and seismic performance through low-cyclic loading tests. Through the study of five precast specimens and two cast-in-situ specimens, it is found that the failure mechanism of the precast specimen is basically the same as that of the cast-in-situ specimen, and there is no slip of the lap steel bars due to anchorage failure, indicating that the connection method is reliable. In addition, the energy dissipation capacity and ductility of the precast specimens are not significantly different from those of the cast-in-situ specimens, and the seismic performance is satisfactory. In conclusion, the above joint connection technology had problems such as high accuracy construction requirements, complex assembly, difficult assembly positioning, and unreliable joint connection quality, and it was difficult to fully utilize the advantages of the assembled concrete structures. Moreover, the existing research was mainly based on the traditional precast shear wall to carry out research. Combining the innovative connection method with reliable connection performance and simple construction with green, lightweight, and energy-saving precast composite walls was the most important issue in the study of the assembled concrete shear wall structures. Therefore, it is vital to further explore the assembled precast concrete shear walls in the aspects of green building materials, lightweight components, and simplified connections.

A novel structure-insulation integrated precast concrete composite wall connected by tooth groove and grouted sleeve was proposed by combining the precast composite wall with novel horizontal joint connection technology. However, the connection performance of the horizontal joint and the seismic performance of the precast concrete composite wall connected by tooth groove and grouted sleeve under low-cyclic reversed load were still unclear at present. Consequently, two full-scale precast concrete composite walls connected by tooth groove and grouted sleeve were prefabricated and tested. Firstly, the specimen design and reinforcement of the precast concrete composite wall, the connection structure design of tooth groove and grouted sleeve connection, and the construction and assembly process, loading mode, test setup, and measuring point arrangement of the specimens are elaborated in detail. Secondly, the crack development process and failure mode of precast concrete composite wall connected by tooth groove and grouted sleeve are described, and the hysteresis curve, skeleton curve, stiffness, displacement ductility, energy dissipation, and reinforcement strain of the specimens are studied and discussed comprehensively. Finally, four conclusions are put forward. The main purpose of this paper is to verify the feasibility and reliability of the precast concrete composite wall with this innovative connection method and investigate the seismic performance of the specimens subjected to low-cyclic reversed load. It was expected that the experimental results of this study could supply a basis for the design and practical application of the horizontal joint of the precast concrete composite wall connected by tooth groove and grouted sleeve.

## 2. Experimental Program

### 2.1. Test Specimens

In order to verify the feasibility and reliability of the innovative precast concrete composite wall connected by tooth groove and grouted sleeve and to explore the connection performance of the horizontal joint and the seismic performance of the wall under low-cyclic reversed loading, two full-scale precast concrete composite wall specimens were designed and prefabricated, numbered PW1 and PW2, respectively. The details of the specimens are shown in Table 1.

**Figure 1:**
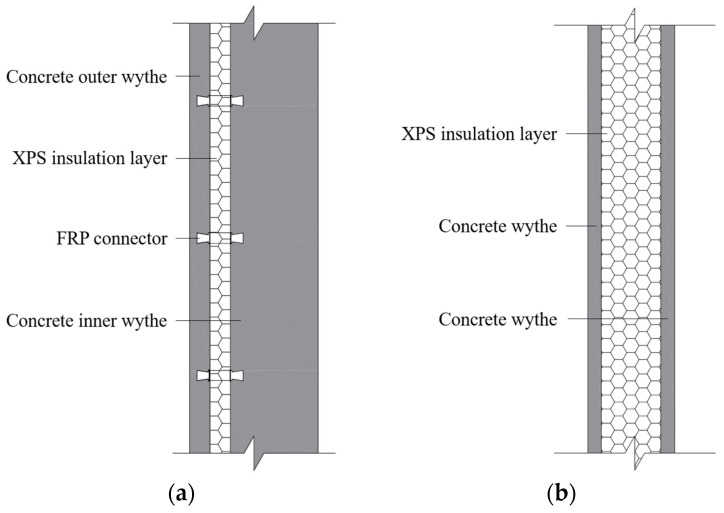
Configuration of precast concrete composite wall: (**a**) PW1 with external insulation layer; (**b**) PW2 with sandwich insulation layer.

PW1 is composed of two concrete wythes and an extruded polystyrene (XPS) foam board insulation layer, where the concrete wythes are connected by fiber-reinforced polymer (FRP) connectors passing through the XPS insulation layer with a spacing of 600 mm. Taking into account the thickness requirements of the insulation layer of residential buildings in Hunan, the thickness of the XPS insulation layer is set to 40 mm, and the steel fabric is embedded in a 40 mm thick concrete outer wythe to prevent the XPS insulation layer from detachment failure prematurely. And the thickness of the inner concrete wythe is 200 mm, which is the structural layer of the precast concrete composite exterior wall. As the exterior walls of buildings usually need to reserve doors and windows in practical engineering, an opening with a size of 1800 × 1500 mm was reserved on PW1. In order to take into account the thermal insulation performance of the wall panel and weaken the stiffness and self-weight of the wall, the precast concrete composite interior wall PW2 is hollowed out to optimize the seismic performance. That is, the surrounding area is concrete rib-beam and rib-column, and the central area (2400 × 1400 mm) consists of a 120 mm-thick XPS insulation layer inserted between two 40 mm-thick concrete wythes equipped with steel fabric. The configuration of the precast concrete composite wall PW1 and PW2 is shown in Figure 1.

**Figure 2:**
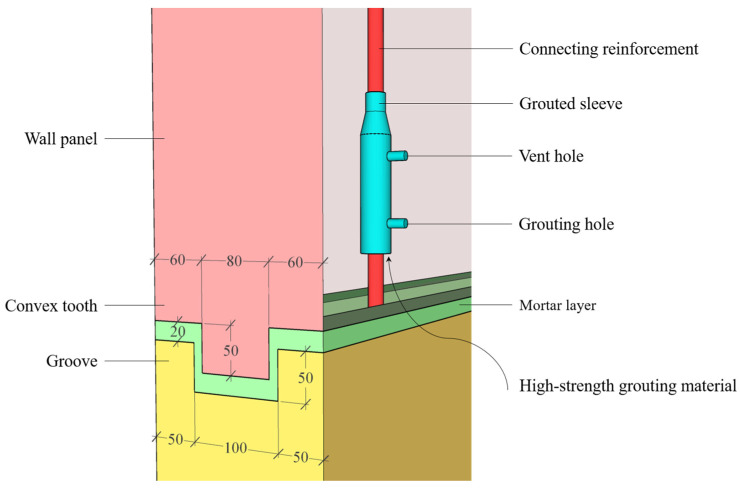
The details of the horizontal joints (Unit: mm).

Each precast concrete composite wall specimen is composed of a wall panel and a foundation, and the horizontal joints are connected by tooth groove and grouted sleeve. As shown in Figure 2, the convex tooth and groove are preformed at the bottom of the precast concrete composite wall panel and the top surface of the foundation, and a 20 mm thick mortar layer is poured in the horizontal direction for connection. In this paper, the geometrical dimensions of the convex tooth and groove are 80 × 50 mm and 100 × 50 mm, respectively. Such a tooth groove configuration can be accurately positioned during the assembly process, can improve the assembly efficiency, and can alleviate the current problems such as easy leakage of flat direct joints in most prefabricated buildings [29]. In addition, four connecting reinforcements with a diameter of 20 mm (HRB400) protrude from the foundation in the vertical direction and are anchored in the grouted sleeves embedded in the wall panel by high-strength grouting material. The lap length of the connecting reinforcement is conservatively taken as 1200 mm.

**Figure 3:**
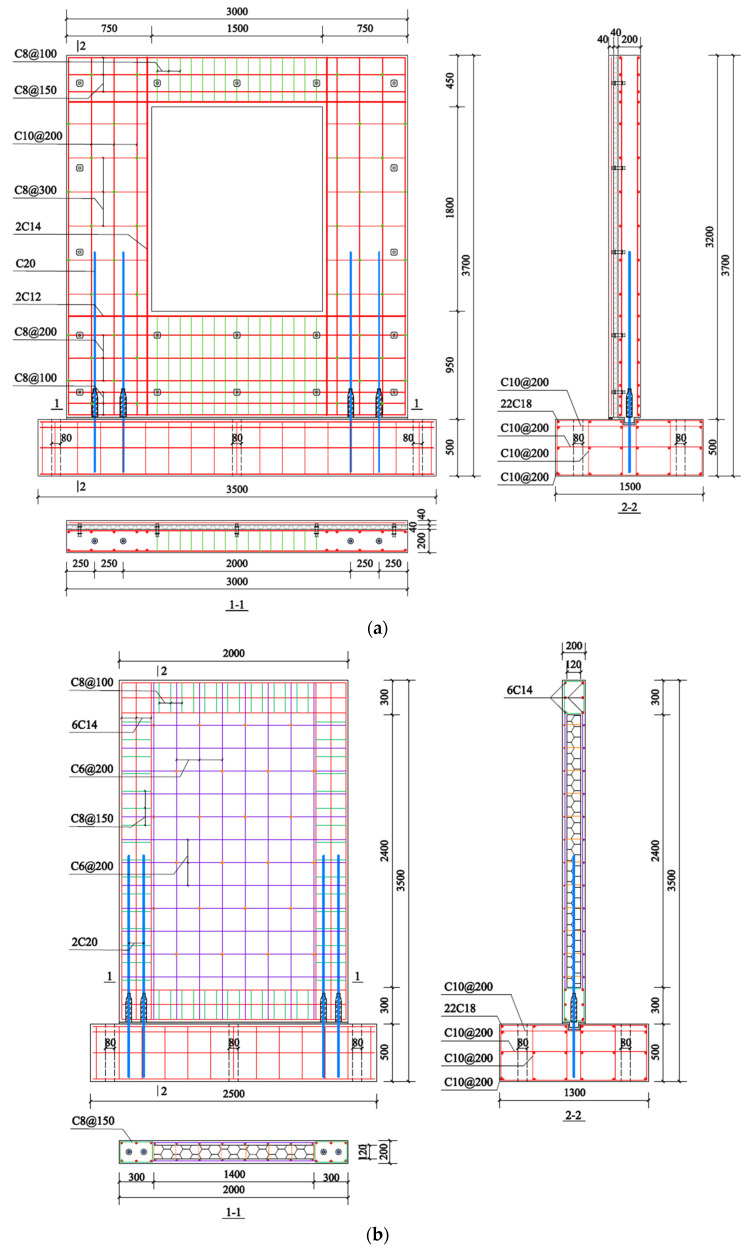
Reinforcement of specimens (Unit: mm): (**a**) PW1; (**b**) PW2.

The type of concrete used in this study is cement concrete, using ordinary P.O 42.5 Portland cement, and its main chemical composition includes CaO, SiO_2_, Al_2_O_3_, Fe_2_O_3_, SO_3_, MgO, Na_2_O, K_2_O, etc. The concrete strength of the wall panel and foundation is C30 and C40, respectively. The precast concrete composite wall geometry and reinforcement are depicted in Figure 3. The specimens are equipped with HRB400 reinforcements, which are represented by the letter “C”. Due to the weak location at the PW1 opening, which is prone to cracking due to stress concentration, a horizontal reinforcement with a diameter of 12 mm and a vertical reinforcement with a diameter of 14 mm are arranged at the opening to strengthen it. Under the action of an earthquake, the coupling beam above the opening should have sufficient strength to resist the external load, so the longitudinal reinforcement of the coupling beam is arranged in double rows of 6C10 to ensure sufficient stiffness without premature failure, and stirrups with a diameter of 8 mm are densely arranged with a spacing of 100 mm. The horizontal reinforcement of the wall limbs on both sides is C8@300, while the longitudinal reinforcement is C10@200. Moreover, 12C8 is adopted for the longitudinal reinforcement of the wall under the window, and the longitudinal reinforcement is densely arranged within the range of 300 mm of the horizontal joint at the bottom of the wall panel. The dimension of the rib-beam and rib-column of PW2 is 300 × 300 mm, and the horizontal and longitudinal reinforcements are 6C14. C8 stirrups are reinforced in the rib-beam and rib-column with a spacing of 100 and 150 mm, respectively. In addition, a steel fabric with a diameter of 6 mm and a spacing of 200 mm is installed in the concrete wythes of PW2 to enhance, which can prevent concrete from cracking prematurely under loading.

### 2.2. Materials

In this study, concrete, mortar, and high-strength grouting material were used. According to the specification requirements of GB/T 50152-2012 [46] and JGJ/T70-2009 [47], the concrete, mortar, and high-strength grouting material samples were reserved and cured for 28 days, and then the compressive strength was measured. The test results are shown in Table 2. In addition, monotonic axial tension tests of HRB400 reinforcements were conducted to obtain the mechanical properties, as listed in Table 3.

### 2.3. Construction Process

The precast concrete composite wall panel and foundation of this test were prefabricated separately in the factory. Firstly, the reinforcements were polished and the strain gauges were pasted at the polished position, and then wrapped with epoxy resin to prevent the strain gauges from being damaged during the pouring and installation process. Secondly, the formwork was set up, the opening was reserved, and then the tied reinforcement cages were placed in the formwork. After casting the concrete inner wythe, the XPS insulation layer was arranged above the concrete and the steel fabric of the concrete outer wythe was laid on it. Before the initial setting of the concrete structural layer, the FRP connectors were anchored through the XPS insulation layer, and the concrete outer wythe was poured. When the specimens were fabricated and cured to a certain strength, they were transported to the National Engineering Research Center of High-speed Railway Construction Technology of Central South University for assembly.

**Figure 4:**
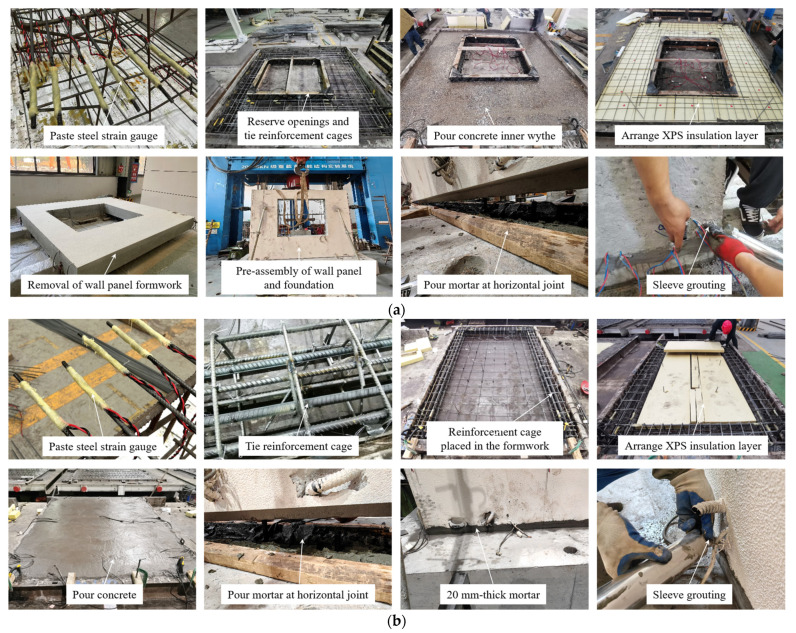
Construction process of specimens: (**a**) PW1; (**b**) PW2.

The assembly process of PW1 and PW2 was basically the same. Firstly, the wall panel and the foundation were pre-assembled and supported in place with the inclined support, and then the tooth groove configuration at the horizontal joint was connected by mortar. After 7 days of curing, the sleeves were grouted with high-strength grouting material until the high-strength grouting material flowed out of the vent hole of the sleeve. The construction and assembly process of specimens is shown in Figure 4.

### 2.4. Test Setup

**Figure 5:**
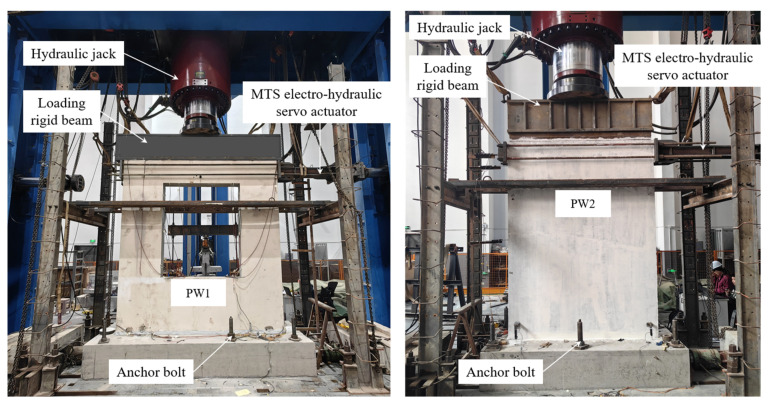
Test setup.

**Figure 6:**
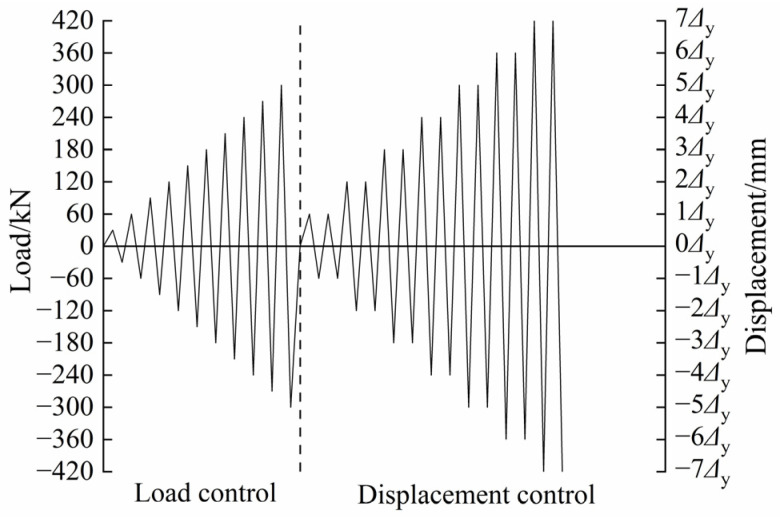
Loading mode of low-cyclic reversed loading test.

The low-cyclic reversed loading test was performed on the multi-function testing system with a capacity of 20,000 kN in the National Engineering Research Center of High-speed Railway Construction Technology of Central South University, and Figure 5 shows the test loading device. The horizontal low-cyclic reserved load is applied by the MTS electro-hydraulic servo actuator, which is located 150 mm from the top surface of the wall panel, and the vertical axial force is applied by loading rigid beams and hydraulic jacks. In the test, the vertical axial load corresponding to the axial compressive ratio of 0.1 is first applied (the axial compressive ratio can be calculated according to Equation (1)), and then the horizontal low-cyclic reversed load is implemented on specimens. Before yielding, the horizontal load is controlled by load, and the load of each grade is 30 kN, which increases successively. When the specimens occur a notable displacement (the connecting reinforcement of the wall panel normally reaches the yield strain), the loading system is transformed into displacement control. The load of each grade is taken as the yield displacement, and each grade is cycled twice, the loading process is depicted in Figure 6 [48]. When the horizontal load eventually decreases to about 85% of the maximum load, the test is stopped, and the value of this displacement is defined as the ultimate displacement.

*u* = *N*/*f*_c_·*A* (1)

where *u* is the axial compressive ratio, *N* is the vertical axial load, *f*_c_ is the compressive strength of concrete, and *A* is the total cross-section area of the wall panel.

### 2.5. Content of Measurement

**Figure 7:**
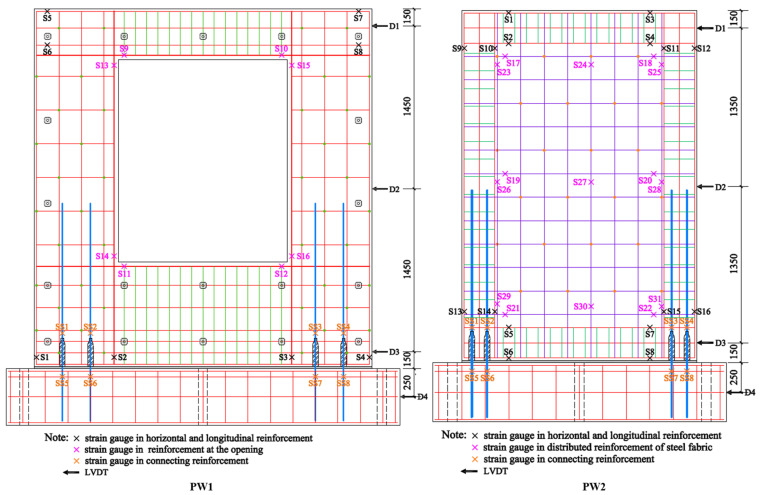
Arrangement of measuring points (Unit: mm).

The horizontal force, displacement, and strain of reinforcements at the key position of the specimens were measured during the test. The horizontal force is measured by the force sensor of the MTS electro-hydraulic servo actuator itself. The displacement is recorded by high-precision linear variable differential transformers (LVDTs), which are labeled D1, D2, D3, and D4. In addition, the strain of reinforcements at key positions is measured by strain gauges. The measurement points of strain and displacement are shown in Figure 7.

## 3. Results and Discussion

### 3.1. Failure Mode

**Figure 8:**
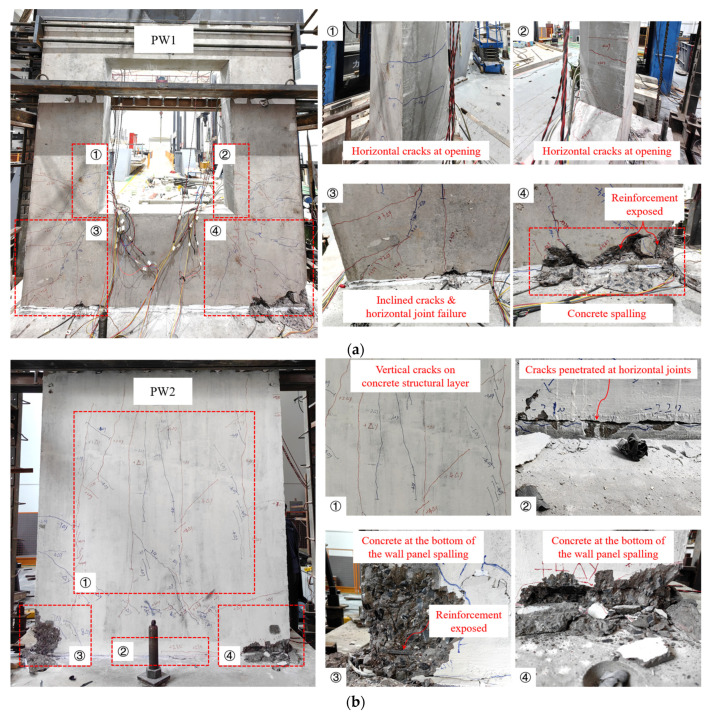
Failure mode of specimens: (**a**) PW1; (**b**) PW2.

The failure mode and distribution of cracks in the specimens are shown in Figure 8. The horizontal cracks were first initiated around the opening of the specimen PW1. As the test progressed, short horizontal cracks gradually developed and the number of cracks increased. The reason may be that the concrete strength was slightly lower than other positions of the wall panel due to the problems of uneven pouring and uncompacted vibration at the opening, so the opening became a weak part and was prone to cracking under load [17]. At the same time, inclined cracks occurred and expanded on the wall panel below the opening, and the width of the cracks became larger with the increase in load. When the loading system was transformed into displacement control, the connecting reinforcements were partially yielded and the cracks near the opening increased and expanded significantly. Additionally, it can be observed that the horizontal joint between the wall panel and the foundation was cracked along with slight fragmentation of concrete. However, it was worth noting that the XPS insulation layer always maintained good contact with the concrete without relative displacement. Eventually, the concrete at the wall toes peeled off in a large area and the specimen was destroyed.

For the specimen PW2 filled with a 120 mm thick XPS insulation layer, it was observed that small cracks first occurred near the junction of the rib-beam, rib-column, and the XPS insulation layer. With the increase in load, these cracks gradually extended and developed into short cross-diagonal cracks. Subsequently, a large number of vertical cracks appeared in the concrete wythes of the XPS insulation layer area of the wall panel, extending from the junction of the rib-beam and the XPS insulation layer to the lower junction. The reason was found to be that the thickness of the concrete wythes of the specimen PW2 is 40 mm, which is relatively thin. It caused the concrete wythes to become a weak area under vertical axial load and horizontal cyclic reversed load, and it became stressed before the overall wall panel, resulting in the rapid development of vertical cracks. During this process, the tooth groove connection at the horizontal joint cracked and it continued to expand and penetrate until failure. When the cracks at the horizontal joints were completely penetrated, the concrete at the wall toes began to peel off, and the inclined cracks at the corner of the wall further developed and the width increased significantly. Finally, the concrete at the corners on both sides of the wall panel was severely spalled and damaged, accompanied by partial exposure of reinforcements.

In summary, the failure modes of PW1 and PW2 were both horizontal joint cracking and penetration, and the concrete at the wall toes was crushed and spalled off. Although the horizontal joints were penetrated, the wall panel did not show obvious uplift, indicating that the connecting reinforcements effectively transferred the stress, and the tooth groove and grouted sleeve connection performance was good. In addition, the XPS insulation layer and the concrete wythes of the precast concrete composite wall always maintain good bond performance under load, and the precast concrete composite wall shows good integrity as a whole.

### 3.2. Hysteretic Curve and Skeleton Curve

**Figure 9:**
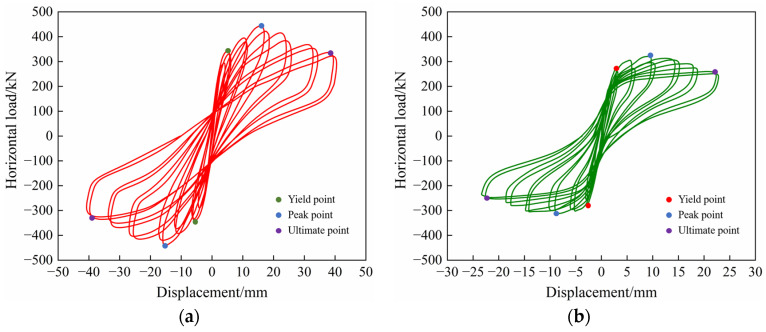
Hysteretic curves: (**a**) PW1; (**b**) PW2.

The area of the hysteretic curve for each grade of the horizontal load and displacement reflects the deformation capacity, the energy dissipation capacity, and the ductility of the specimens during low-cyclic reversed loading [49]. The hysteretic curves of precast concrete composite walls connected by tooth groove and grouted sleeve PW1 and PW2 are plotted in Figure 9. PW1 and PW2 experience four stages of cracking, yielding, peaking, and failure under low-cyclic reversed loading. Overall, the shapes of the hysteretic curves are similar. It can be seen from Figure 9 that the specimens are dominated by elastic deformation before cracking, and the hysteretic curves basically change linearly. In the initial stage of cracking, the stiffness of the specimen is slightly degraded, and the area surrounded by the hysteretic loop is small and shuttle-shaped. With the increase in load, the specimens gradually enter the elastic-plastic stage. At this time, the area surrounded by the hysteretic loop increases obviously, and the hysteretic shape is fuller. As the load continues to increase, the number of cracks gradually increases, and the hysteretic curves develop from shuttle-shaped to bow-shaped. It can be observed that the hysteretic curves of both PW1 and PW2 show a pinching phenomenon, which is mainly attributed to the influence of the degradation of the bond behavior between the concrete and the connecting reinforcements caused by the penetration of cracks at the horizontal joints of the specimens. In addition, it can be found that in the same loading stage, after the precast concrete composite wall specimens yield, the peak load point of the second hysteretic loop is usually lower than the first hysteretic loop, which is mainly due to the decrease in the strength of the specimens caused by the accumulation of concrete internal damage [50]. After the peak point, the bearing capacity of the specimens decreases slowly with the increase in displacement, showing good ductility. In summary, the hysteretic loops of precast concrete composite exterior wall and interior wall are relatively full and the energy dissipation capacity is good. Moreover, the XPS insulation layer and concrete wythes show excellent cooperative working performance, the overall mechanical performance of the specimens is good, and the plasticity of the wall panel is sufficiently developed.

**Figure 10:**
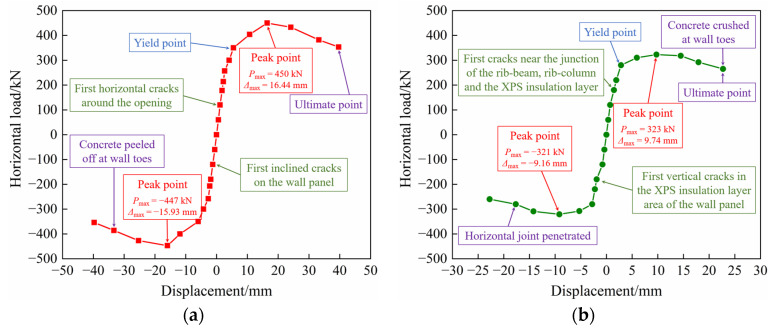
Skeleton curves: (**a**) PW1; (**b**) PW2.

The skeleton curve is the envelope of the hysteretic curve, and the skeleton curves of precast concrete composite walls connected by tooth groove and grouted sleeve are delineated in Figure 10, and the characteristic load and corresponding displacement of PW1 and PW2 derived from the skeleton curves are listed in Table 4 (the data in Table 4 are the average of the absolute values of the data in the two directions). The cracking load is defined as the horizontal load corresponding to the occurrence of the first crack during the loading process, the yield load is defined as the horizontal load corresponding to the obvious plastic deformation of the specimen, the peak load is the maximum horizontal load value of the specimen during the loading process, and the ultimate load is defined as 85% of the peak load. It can be found that all skeleton curves were approximately straight lines before cracking. After cracking, the slopes of the skeleton curves gradually decrease and it becomes more pronounced when the specimens yield. When the peak load point is reached, PW1 and PW2 enter the plastic development stage, and the skeleton curves descend steadily until failure. Compared with PW1, the skeleton curve of PW2 decreases more gently. It is worth noting that from the perspective of bearing capacity, the yield load, peak load, and ultimate load of PW1 increase by 29.5%, 38.6%, and 33.2% compared with PW2, respectively. The reason is that the precast concrete composite exterior wall adopts the XPS external insulation form connected by FRP connectors, whose concrete structural layer is thicker (200 mm), and the integrity of the specimen is better. Therefore, the precast concrete composite exterior wall PW1 shows a higher bearing capacity.

### 3.3. Stiffness Degradation

**Figure 11:**
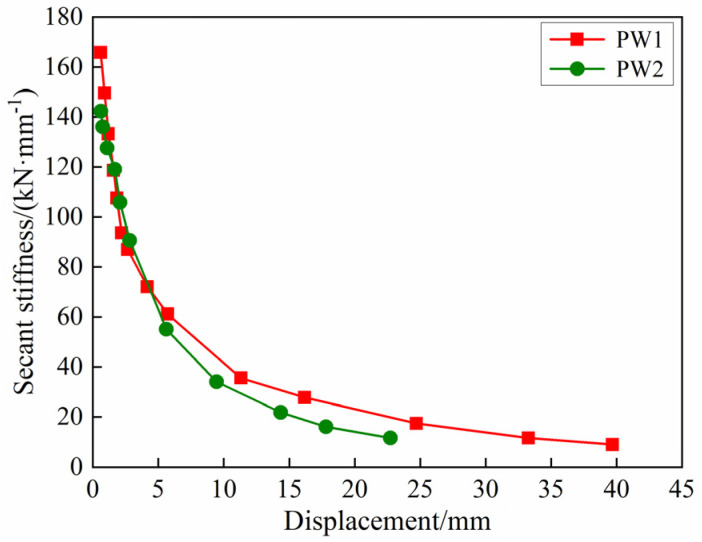
Secant stiffness degradation.

In this paper, secant stiffness is used to measure the stiffness degradation of the precast concrete composite wall specimens, and the secant stiffness can be calculated by Equation (2). The stiffness degradation curves of PW1 and PW2 based on the skeleton curves are shown in Figure 11. It can be seen that the stiffness degradation of PW1 and PW2 is a typical precast concrete wall stiffness degradation mode, that is, the stiffness decreases rapidly in the initial stage, and the stiffness degradation gradually tends to be gentle after yielding. This is because the generation and development of cracks are mainly concentrated before yielding, only a small number of new cracks appear or expand after yielding, and the specimens mainly rely on the tensile cracking and crushing of concrete to dissipate energy. The stiffness of precast concrete composite exterior wall specimen PW1 is slightly larger than that of precast concrete interior wall specimen PW2, which indicates that the precast concrete composite exterior wall with a thicker concrete structural layer possesses strong lateral stiffness. Overall, the stiffness degradation of the precast concrete composite wall connected by tooth groove and grouted sleeve is not different from that of other precast concrete walls [17]. This shows that the design of a precast structure-insulation integrated composite wall connected by tooth groove and grouted sleeve has a certain rationality.

(2) $$K_{i}=\frac{\left|+P_{i}\right|+\left|-P_{i}\right|}{\left|+\Delta_{i}\right|+\left|-\Delta_{i}\right|}$$

where *K_i_* is the secant stiffness, +*P_i_* and −*P_i_* are the positive and negative peak loads of the *i*th cycle, respectively, +Δ*_i_* and −Δ*_i_* are the displacement corresponding to the positive and negative peak load of the *i*th cycle, respectively.

### 3.4. Displacement Ductility and Deformability 

In this paper, the displacement ductility coefficient *μ* is used to measure the ductility of the specimen, which can be calculated by dividing the ultimate displacement by the yield displacement (Equation (3)) [51], and the calculation results of displacement ductility coefficient are shown in Table 4. The displacement ductility coefficients of precast concrete composite exterior wall PW1 and precast concrete composite interior wall PW2 are 6.9 and 7.5, respectively. Both of them are of good ductility and strong deformation ability. The reason is that the dislocation of the tooth groove connection interface causes a large displacement of the wall panel and the deformation of the connecting reinforcements at the horizontal joint is large, resulting in the ductility of PW1 and PW2 greater than the displacement ductility coefficient 3–4 required by the reinforced concrete seismic structures. Therefore, it can be shown that the precast concrete composite wall with tooth groove and grouted sleeve has good ductility performance.

*μ =* Δ_u_/Δ_y_ (3)

where *μ* is the displacement ductility coefficient, Δ_u_ is the ultimate displacement, Δ_y_ is the yield displacement.

### 3.5. Energy Dissipation Capacity

**Figure 12:**
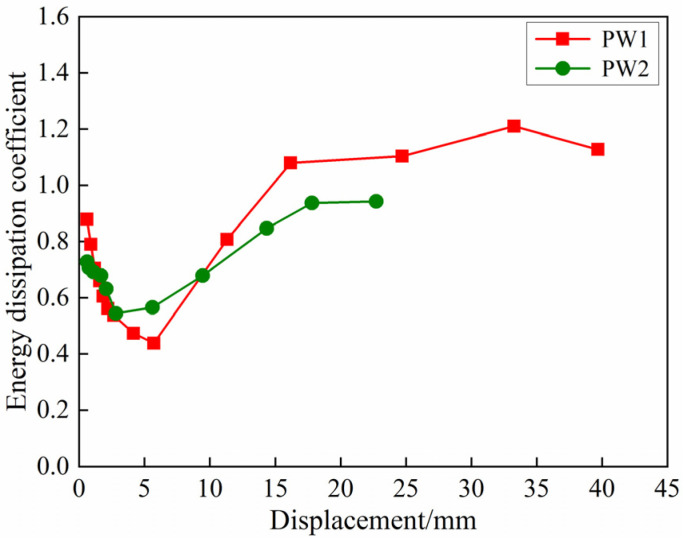
Energy dissipation coefficient.

The energy dissipation capacity is usually evaluated by the energy dissipation coefficient, which is the ratio of the dissipated energy of the specimen to the elastic potential energy of the specimen at the peak load of each hysteretic loop [52], and the calculation formula is shown in Equation (4). The energy dissipation coefficient of PW1 and PW2 is shown in Figure 12. It can be found that before yielding, the energy dissipation capacity of the precast concrete composite wall connected by tooth groove and grouted sleeve decreases rapidly and reaches the minimum at the yield point (Δ_PW1_ is about 6 mm, Δ_PW2_ is about 3 mm). The reason is that the cracks appear on the wall panel and continue to extend in the initial stage of loading, which dissipates part of the energy. Thereafter, as the connecting reinforcements of the specimens participate in plastic energy dissipation and joint stress with concrete, the energy dissipation capacity of the specimens gradually enhances [53]. In the failure stage, the energy dissipation coefficients of PW1 and PW2 reach about 1.2 and 0.9, respectively. It can be seen from the comparison that the energy dissipation capacity of PW2 is slightly lower than that of PW1. The reason may be that the deformation of the edge connecting reinforcement of the specimen PW2 is large, and more energy is dissipated during the test.

**Figure 13:**
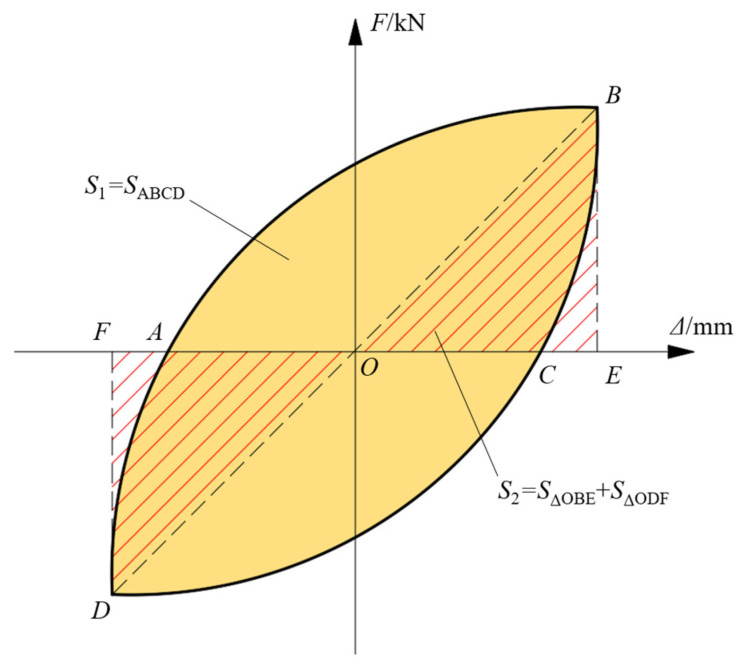
Hysteretic loop energy dissipation diagram.

*E* = *S*_1_/*S*_2_ (4)

where *E* is the energy dissipation coefficient, *S*_1_ is the area surrounded by a single hysteretic loop, *S*_2_ is the area surrounded by triangles, and the calculation diagram of *S*_1_ and *S*_2_ is shown in Figure 13.

### 3.6. Reinforcement Strain

#### 3.6.1. Connecting Reinforcement Strain

**Figure 14:**
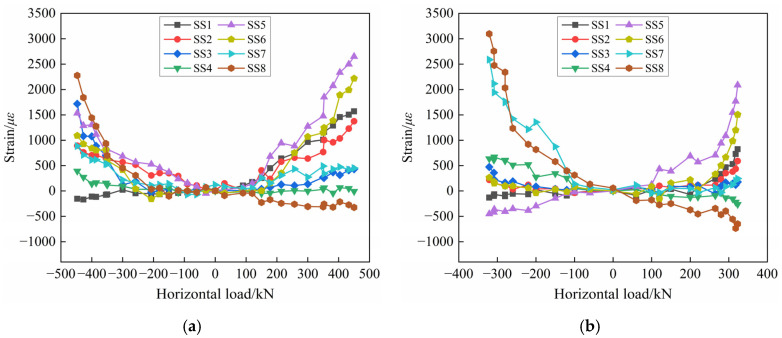
Strain diagram of connecting reinforcement: (**a**) PW1; (**b**) PW2.

The wall panels of the precast concrete composite wall specimens are connected to the foundation by the connecting reinforcement, so the strain gauges are set up at the upper and lower positions of the sleeve to obtain the stress of the connecting reinforcement at the horizontal joint. The connecting reinforcement strain of PW1 and PW2 is shown in Figure 14. With the increase in the horizontal load, the connecting reinforcement strains grow gradually, and some of them can reach yielding finally. However, some of the connecting reinforcement strains are still lower than the yield strain when the specimen is destroyed, indicating that the material strength is not fully utilized. Therefore, the diameter of the connecting reinforcement can be appropriately reduced. It is worth noting that the strain direction of the outermost connecting reinforcements of the PW1 and PW2 wall panels is opposite to that of the other three connecting reinforcements under the same loading stage. For example, under the positive load, the measuring points SS1, SS2, and SS3 are opposite to SS4, and SS5, SS6, and SS7 are opposite to SS8. It shows that the two connecting reinforcements on the outer side bear the tensile and compressive load at the same time, while the two connecting reinforcements on the inner side are basically in the tensile state. It is worth noting that it can be seen from Figure 14 that the strain variation of measuring points SS7 and SS8 of PW2 is obviously different from that of PW1. The reason is that under the action of vertical axial load and horizontal low-cyclic reversed load, the phenomenon of concrete spalling damage at the corner of the wall is more serious because the thickness of PW2 (200 mm) is smaller than that of PW1 (280 mm), and the connecting reinforcements at this position bear more stress. Therefore, the strain values of measuring points SS7 and SS8 of PW2 are significantly larger than those of PW1. It can be seen from the figure that the strain at the lower end (measuring points SS5, SS6, SS7, and SS8) of the connecting reinforcement is greater than that at the upper end (measuring points SS1, SS2, SS3, and SS4), indicating that the connecting reinforcement can achieve effective stress transfer between the wall panel and foundation, and the connection performance and stress transfer mechanism of the tooth groove and grouted sleeve connection horizontal joint are reliable.

#### 3.6.2. Horizontal and Longitudinal Reinforcement Strain

**Figure 15:**
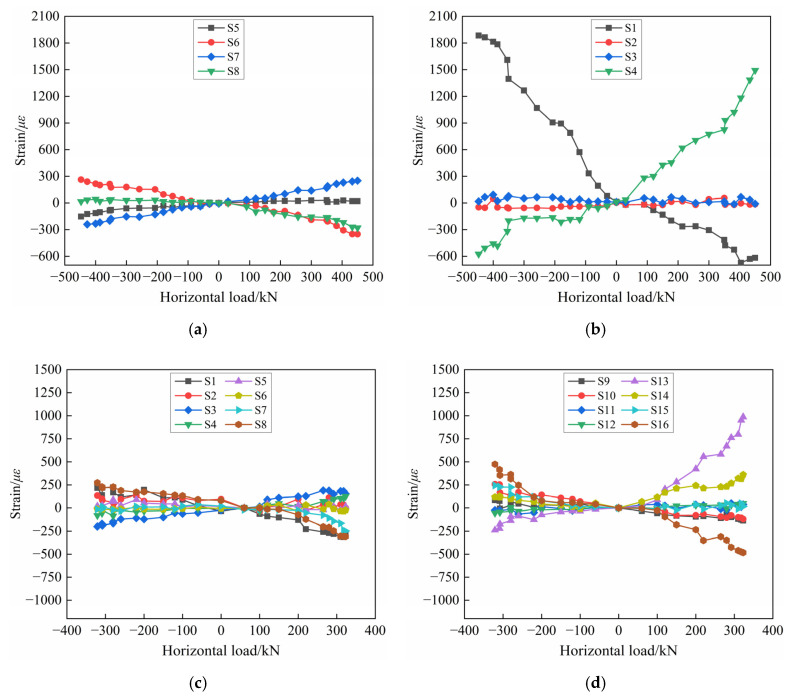
Strain diagram of horizontal and longitudinal reinforcement: (**a**) Horizontal reinforcement of PW1; (**b**) Longitudinal reinforcement of PW1; (**c**) Horizontal reinforcement of PW2; (**d**) Longitudinal reinforcement of PW2.

The strains of the horizontal and longitudinal reinforcement of PW1 and PW2 are shown in Figure 15. From Figure 15a, it can be seen that the strains of the horizontal reinforcement in the coupling beam increase with the increase in the horizontal load, but the strain value is constantly less than 400 με, which is far less than the yield strain. This shows that the main role of the PW1 coupling beam is to connect the wall limbs on both sides to bear stress cooperatively, and the load on the horizontal reinforcements is comparatively small. In addition, it can be known from Figure 15b that the strains of the longitudinal reinforcements of the wall panel increase regularly with the increase in the load. Under the same load level, the strain growth rate of the outer longitudinal reinforcements is faster, and it basically reaches yielding in the ultimate stage, while the strain value of the inner longitudinal reinforcement does not change much, basically within 200 με. This indicates that when PW1 is close to failure, the outer longitudinal reinforcements of the wall panel bear part of the horizontal load, but most of the load is borne by the connecting reinforcements at the horizontal joint, as shown in Figure 14a. Furthermore, it is also found that the strain directions of the longitudinal reinforcements on both sides of the PW1 wall panel are opposite, which means one side of the longitudinal reinforcements is subjected to tension or compression, and at the same time, another side of the longitudinal reinforcements are under compression or tension, for example, the measurement points S1 and S4 in PW1.

It is found from Figure 15c,d that the strains of the horizontal and longitudinal reinforcements in the PW2 rib-beam and rib-column gradually increase as the test progresses, but neither reach the yielding. The strain values of the horizontal reinforcements in the rib-beam are basically within 300 με, while the strain values of the longitudinal reinforcements in the rib-column are mainly within 700 με. This is because the horizontal joint between the PW2 wall panel and the foundation cracks and eventually penetrates with the increase in load, which becomes the main stress point of specimen PW2. Therefore, the horizontal and longitudinal reinforcements in the rib-beam and the rib-column are subjected to relatively small loads, whose strain does not reach the yield strain in the ultimate stage. At this time, the connecting reinforcements at the horizontal joint have partially yielded (Figure 14b), so the flexural capacity of the specimen PW2 is principally provided by the connecting reinforcements at the horizontal joint.

It is noteworthy that there are obvious differences in the longitudinal reinforcement strain changes in PW1 and PW2, as shown in Figure 15b,d. The strain values of the measuring points S1 and S4 of PW1 are basically close to the yield strain, while in addition to the larger strain value of measuring point S13, all other measuring points of PW2 are within 500 με. The reason is that for the precast concrete composite wall connected by tooth groove and grouted sleeve, the horizontal load is mainly borne by the connecting reinforcements at horizontal joints, and with the penetration failure of the tooth groove joints of the specimen, the concrete at wall toes crack and peel off, and the wall panel is slightly uplifted. The longitudinal reinforcements on both sides of the PW1 wall panel also participate in energy dissipation and basically reach yield, so the measuring points S1 and S4 of the longitudinal reinforcements near the horizontal joint of PW1 increase significantly with the increase in load. While the arrangement of the longitudinal reinforcement measuring points of PW2 is about 350 mm away from the horizontal joint, which is much larger than that of PW1 (50 mm away from the horizontal joint), the strain change in the longitudinal reinforcements of PW2 is not as significant as that in PW1.

#### 3.6.3. Constructional Reinforcement Strain at the Opening

**Figure 16:**
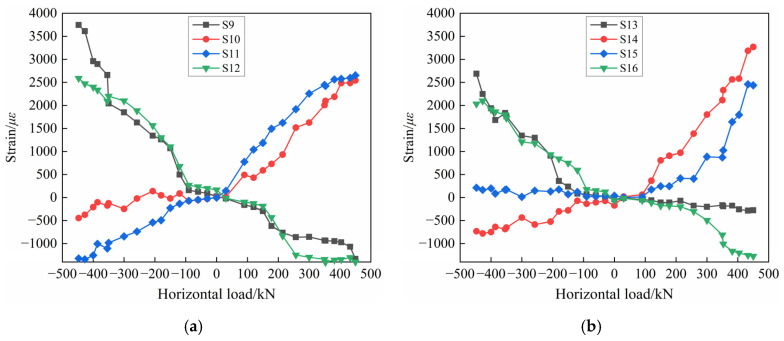
Strain diagram of constructional reinforcement of PW1: (**a**) Horizontal constructional reinforcement; (**b**) Longitudinal constructional reinforcement.

The strains of constructional reinforcements at the opening of PW1 (the measurement points of the horizontal constructional reinforcements numbered S9–S12 and the measurement points of the longitudinal constructional reinforcements numbered S13–S16) are shown in Figure 16. Under the low-cyclic reversed loading, the strains of the constructional reinforcements at the opening increase continuously. When the ultimate stage is reached, the strains of the horizontal and longitudinal constructional reinforcements have reached yielding, which suggests that the stress concentration is prone to occur at the opening of the wall panel. And it is easy to cause the development of concrete cracks near the opening, which is consistent with the experimental phenomena described in Section 3.1. By comparing Figure 16a,b, it can be found that the strain change trend of horizontal and longitudinal constructional reinforcements is roughly consistent, but the strains of horizontal constructional reinforcements (up to about 3800 με) are slightly larger than that of longitudinal constructional reinforcements (up to about 3400 με). The reason may be that the horizontal constructional reinforcements at the opening are subjected to certain bending and shearing under the combined action of vertical axial force and horizontal low-cyclic reversed load.

#### 3.6.4. Distributed Reinforcement Strain of Steel Fabric

**Figure 17:**
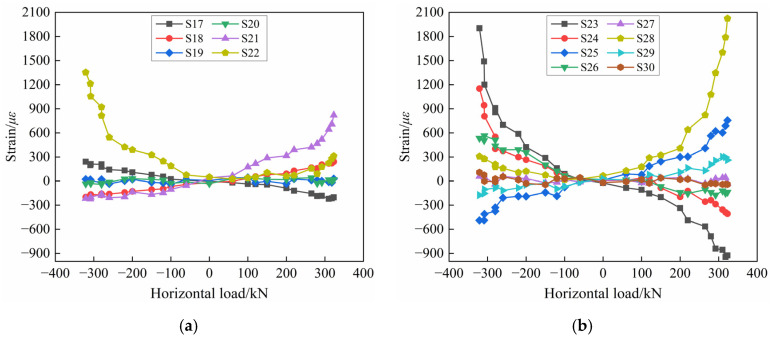
Strain diagram of distributed reinforcement of PW2: (**a**) Horizontal distributed reinforcement; (**b**) Longitudinal distributed reinforcement.

The strains of the distributed reinforcement in the steel fabric of PW2 (the strain measurement points of the horizontal and longitudinal distributed reinforcement of the steel fabric are arranged and numbered as shown in Figure 7) are shown in Figure 17. It can be seen from Figure 17a that the horizontal reinforcement strain of the PW2 steel fabric is always much smaller than the yield strain, and the strain of measuring points S21 and S22 is larger, which may be due to the fact that the measuring points S21 and S22 are close to the concrete spalling area at the wall toes, resulting in a slightly larger strain than other measuring points of the steel fabric. It can be found from Figure 17b that the longitudinal reinforcement strain of the PW2 steel fabric is partially close to yielding in the ultimate stage. The strain values of the measuring points S23, S24, S25, and S28 are larger, and the reason for this is that the concrete wythes and XPS insulation layer of specimen PW2 are jointly subjected to stress during the whole test, while the 40 mm-thick concrete wythe is thinner, and the longitudinal reinforcement of the steel fabric in the concrete wythe bears most of the load, resulting in concrete cracking from the top. It is manifested as long vertical cracks occurring on the concrete wythe within the XPS insulation layer area under vertical axial load and horizontal low-cyclic reversed load, and the short cross-diagonal cracks appear near the junction of the rib-beam, rib-column, and XPS insulation layer, which is consistent with the test phenomenon shown in Figure 8b of Section 3.1.

## 4. Conclusions

In this paper, a novel precast concrete composite wall with XPS foam board as an insulation layer connected by tooth groove and grouted sleeve was proposed, and the feasibility and reliability of this novel connection method were verified and the seismic behavior was explored through the low-cyclic reversed loading tests of two full-scale specimens. The conclusions can be drawn as follows:

(1) A novel joint connection method was proposed wherein the convex tooth and groove were reserved in the horizontal direction, mortar was used for connection, the sleeve and connecting reinforcements were embedded in the vertical direction, and high-strength grouting material was used for grouting. This novel connection method can realize the assembly only by pouring mortar and grouting, which had the advantages of no template, easy positioning, convenient construction, and high assembly rate.
(2) The structure-insulation integrated prefabrication of the precast concrete composite wall with XPS foam board as an insulation layer can reduce the self-weight of the wall, facilitate transportation and hoisting, and help solve the problem that the traditional insulation layer is easily falls off. However, the precast concrete composite wall still faces issues such as poor fire resistance and difficulty in preventing isolation from moistening and biological decay during the construction process.
(3) Although the horizontal joints of the precast concrete composite walls cracked and eventually penetrated under the vertical axial load and horizontal low-cyclic reversed load, connecting reinforcements can always effectively transfer stress without bond failure. Thus, the novel tooth groove and grouted sleeve connection method was reliable. Moreover, the XPS insulation layer and concrete wythes showed satisfactory cooperative working performance, which demonstrated that the precast structure-insulation integrated composite wall connected by tooth groove and grouted sleeve had certain rationality.
(4) The hysteretic curves of the precast concrete composite wall connected by tooth groove and grouted sleeve were full, but the bearing capacity and energy dissipation capacity of PW1 were slightly larger than PW2. Furthermore, the displacement ductility coefficients of both PW1 and PW2 were greater than 6, showing good ductility and deformation capability. On the whole, the seismic performance of the precast concrete composite wall connected by tooth groove and grouted sleeve was satisfactory, and it can be used in practical engineering projects under reasonable design.

## Figures and Tables

**Table 1:** Design parameters of specimens (Unit: mm).

Specimen	Type	Insulation Layer Material	*T*/mm	*T*_i_/mm	*T*_1_/mm	*T*_2_/mm	Opening/mm	*μ*
PW1	Exterior wall	XPS foam board	280	40	40	200	1800 × 1500 × 280	0.1
PW2	Interior wall	XPS foam board	200	120	40	40	/	0.1

Note: *T* is the thickness of the precast composite concrete wall, *T*_i_ is the thickness of insulation layer, *T*_1_ is the thickness of concrete outer wythe, *T*_2_ is the thickness of concrete inner wythe, *μ* is the axial compressive ratio.

**Table 2:** Mechanical properties of concrete, mortar, and high-strength grouting material.

Items	Specimen Size/mm	Load/kN	*f*_c_/Mpa
Concrete-C30	150 × 150 × 150	753.7	33.5
Concrete-C40	150 × 150 × 150	943.4	41.9
Mortar	70.7 × 70.7 × 70.7	244.6	48.9
High-strength grouting material	40 × 40 × 160	146.1	91.3

Note: *f*_c_ is the compressive strength.

**Table 3:** Mechanical properties of reinforcements.

Strength Grade	Diameter/mm	*f*_y_/Mpa	*f*_u_/Mpa	*δ*/%
HRB400	6	420.2	557.3	19.3
HRB400	8	422.7	561.2	19.8
HRB400	10	429.6	569.7	19.9
HRB400	12	436.9	575.3	21.1
HRB400	14	441.2	584.0	21.4
HRB400	18	446.8	609.7	22.6
HRB400	20	453.6	622.5	22.3

Note: *f*_y_ is the yield strength, *f*_u_ is the tensile strength, *δ* is the elongation.

**Table 4:** Characteristic value.

Specimen	*P*_cr_/kN	Δ_cr_/mm	*P*_y_/kN	Δ_y_/mm	*P*_max_/kN	Δ_max_/mm	*P*_u_/kN	Δ_u_/mm	*μ*
PW1	150	1.58	346	5.72	448	15.19	353	39.66	6.9
PW2	180	1.46	267	3.01	322	9.45	265	22.73	7.5

Note: *P*_cr_ is the cracking load, Δ_cr_ is the cracking displacement, *P*_y_ is the yield load, Δ_y_ is the yield displacement, *P*_max_ is the peak load, Δ_max_ is the peak displacement, *P*_u_ is the ultimate load, Δ_u_ is the ultimate displacement, *μ* is the displacement ductility coefficient.

## Data Availability

Data are contained within the article.
